# K1 Antigen Is Associated with Different AST Profile in *Escherichia coli*: A One-Month-Long Pilot Study

**DOI:** 10.3390/microorganisms9091884

**Published:** 2021-09-05

**Authors:** Maelys Proquot, Lovasoa Najaraly Jamal, Chloe Plouzeau-Jayle, Anthony Michaud, Lauranne Broutin, Christophe Burucoa, Julie Cremniter, Maxime Pichon

**Affiliations:** 1 Bacteriology and Infection Control Laboratory, Infectious Agents Department, University Hospital of Poitiers, 86021 Poitiers, France; maelys.proquot@etu.univ-poitiers.fr (M.P.); lovasoa.najaraly.jamal@etu.univ-poitiers.fr (L.N.J.); chloe.plouzeau-jayle@chu-poitiers.fr (C.P.-J.); anthony.michaud@chu-poitiers.fr (A.M.); lauranne.broutin@chu-poitiers.fr (L.B.); christophe.burucoa@chu-poitiers.fr (C.B.); julie.cremniter@chu-poitiers.fr (J.C.); 2 INSERMU1070 Pharmacology of Antimicrobial Agents, Faculty of Medicine and Pharmacy, University of Poitiers, 86021 Poitiers, France

**Keywords:** *Escherichia coli*, K1 antigen, virulence, maternal-fetal infection, antibiotic susceptibility testing, resistance to antimicrobial agents

## Abstract

*Escherichia coli* is responsible for diseases of varying severity. The “K” antigen designates the capsular polysaccharides on the bacterial surface, which are mostly similar to those of highly pathogenic bacteria. The K1 antigen is often found in pathogenic *E. coli*. Aim: While the published studies on the AST profile of K1-positive *E. coli* have focused on pregnant women or newborns, this study aimed to characterize the AST profile of K1-positive *E. coli* independently of the clinical sample of isolation. Over a 4-week-long period, all patients hospitalized/consulting at the Poitiers University Hospital presenting a determined AST on *E. coli* were prospectively included to define their K1-status (Pastorex Meningitis) and to collect the clinical (age/sex) or biological metadata (AST/MIC). Among the 296 included samples, no differential representation was observed between K1 results regarding sample nature. K1-negative results were associated with multiple antibiotic-resistance (12.3% vs. 33.0%; *p* < 0.01). AST phenotypes differed between these groups, with a higher proportion of K1-negativity among resistant strains, especially on β-lactams (ureidopenicillin, 25.8% vs. 14.9%; and ampicillin/inhibitor, 50.0% vs. 26.8%; *p* < 0.05) or quinolone (19.8% vs. 7.0%) and sulfamethoxazole-trimethoprim (30.2% vs. 12.3%) (*p* < 0.01). This study analyzed *E. coli* ASTs in clinical samples of all types, regarding their K1-antigen status.

## 1. Introduction

*Escherichia coli* is physiologically a normal inhabitant of the gastrointestinal tract of healthy humans. Under particular conditions, this bacterium may localize in various tissues and be responsible for very diverse diseases [1]. The term K antigen was introduced by Kauffmann et al. to designate a surface structure [2]. To date, most of the categorized K antigens are capsular polysaccharides, similar in their overall properties to the capsular saccharides of highly pathogenic and invasive bacteria, such as *Neisseria meningitidis*, *Streptococcus pneumoniae* or *Haemophilus influenzae* [3,4,5]. Among virulence factors that enable these bacteria to survive and to grow in its host, K1 capsular polysaccharide is often found in pathogenic *E. coli*. The relation between capsular polysaccharides and invasiveness of *E. coli* was first suggested by Smith and then Kauffman [6,7]. Invasiveness was postulated to be their anticomplementary effect [8,9]. The variability of virulence marker expression influences the characteristics of the bacteria, which can finally lead to different phenotypes and antibiotic susceptibility testing (AST) under laboratory examinations in vitro. Depending on the circumstances, the physiological state of the patient and the virulence factors, *E. coli* is one of the main causative agents of both gastrointestinal and non-gastrointestinal infections [10].

Pathogenic *E. coli* bacteria remain a major source of morbidity and mortality, mainly due to neonatal bacterial meningitis [11]. Some of these diseases can lead to death, and among survivors more than fifty percent present serious neurological conditions (seizure disorders, hydrocephalus, developmental delay and hearing loss). Other clinical diseases are caused by K1 capsular serotype, including urinary tract infection (more than 50%) and bacteremia (approximately ten to fifteen percent of adult *E. coli*-caused bacteremia originate from K1-positive strains) [12].

Even if these mechanisms have been shown to differ from the ones of group B streptococci and *Listeria monocytogenes*, K1 capsule antigen is a key virulence factor in the pathogenesis of *E. coli* meningitis. This factor allows *E. coli* to internalize itself into human brain microvascular endothelial cells via a mechanism requiring host cell actin cytoskeleton and transduction pathways [13]. This internalization and pathophysiological process implicates other virulence mechanisms, such as P-fimbriae and outer membrane protein A. The latter has often been researched when focusing on virulence characterization of *E. coli* [14]. Moreover, *E. coli* K1-positive-containing vacuoles are not fused with lysosomes, thereby allowing *E. coli* to cross the blood–brain barrier as living bacteria, which not only allows it to resist most of the macrophage properties, but also has a bearing on its serum resistance [13].

As for virulence determinants, the expression of antibiotic resistance in *Enterobacteriaceae*, such as *E. coli*, is controlled by different environmental signals [15]. More recently, as the genotypic characterization of *E. coli* has improved, the impact and need for understanding of non-gastrointestinal strains has become ever more evident. Indeed, these strains represent an increasing problem for human health management, especially due to the major incidence of antibiotic resistance, often carried by plasmid in gram-negative bacteria (accounting for 64% of the bacterial strains identified in Indian neonates presenting extended spectrum beta-lactamase) [16,17]. As concerns maternal-fetal infection, most of the studies published to date have focused on the characteristics of vaginal and/or rectal *E. coli* of pregnant woman, in association with a particular AST profile. Neonatal *E. coli* strains have demonstrated resistance to aminopenicillins as high as 100%, 78% and 93% (and 90%, 10% and 28% to aminoglycosides) in developing countries, the USA and Spain respectively [18,19,20,21].

While most of the published studies focusing on the AST profile of K1-antigenic *E. coli* implicated in diseases are focused on pregnant women or newborns and neonates, the present study aimed to characterize the AST profile of K1-antigenic *E. coli* among all identified strains in analyzed samples in both adults and children.

## 2. Materials and Methods

### 2.1. Selection Criteria and Demographic/Clinical Characteristics

All hospitalized or consulting patients at the University Hospital of Poitiers in whom *Escherichia coli* was identified and tested for AST were prospectively included over a four-week period (18 January to 14 February 2021). In all included patients, the first bacterial strains for which an AST was performed were collected (in the case of two different aspects, both were considered for further analyses, including AST and K1 determination). If *E. coli* was identified in two different locations/samples on a particular day, the more severe location was preferred (for example: positive blood culture was considered as a reflection of a condition more severe than positive urine culture).

For all included patients, a clinical record file was completed, leading to an anonymized database that included all clinical (age, gender, hospitalization location and type of sampling) or biological metadata (AST results or MIC determination if available, K1 determination when carried out in routine treatment in the usual management of the patient).

### 2.2. AST Determination

After overnight culture, the susceptibility of *E. coli* strains to antimicrobial agents was analyzed by disc-diffusion (i2a, Montpellier, France) on Muller-Hinton (MH) agar (BioRad, Hercules, CA, USA) (for bacterial strains identified in blood culture) or liquid method on Vitek 2 system (bioMérieux, Marcy-l’étoile, France) (for bacterial strains identified in all samples but blood culture). All analytical processes and threshold determinations were evaluated according to the European Committee on Antimicrobial Susceptibility Testing (CA-SFM/EUCAST—April 2020). For all tested strains, AST was interpreted by a senior medical biologist (C. P-J; A.M.; L.B.; CB; J.C. or M.P), to validate technical results and centralize them in a single database. Following collection of the results, the medical biologist responsible for the study (M.P.) classified the bacterial strains according to their phenotypic mode of resistance.

### 2.3. K1 Phenotypic Determination

After the overnight growth of MH agar (BioRad), bacterial colonies were isolated and then the K1 antigen-status was determined using the Pastorex Meningitis kits (Bio-Rad, CA, USA) according to the manufacturer’s recommendations. Briefly, this test employs latex beads covered by mouse monoclonal antibodies specific to *E. coli* K1. In the presence of the K1 antigen, the latex particles are visually agglutinated when they remain in a homogeneous suspension in the absence of this antigen. Positive control was verified using particles sensitized with the mouse monoclonal antibody specific to *E. coli* K1, and negative control consisted of particles sensitized with IgG immunoglobulins from a non-immunized rabbit.

### 2.4. Statistical Analyses

Statistical analyses were performed with GraphPad Prism version 9.0. Descriptive statistics were used to analyze both parametric and nonparametric data as appropriate. The Fisher exact test was used to compare proportions. An unpaired t-test was used to compare continuous data after validation of the normality distribution of the data using a Shapiro-Wilk test. A *p*-value below 0.05 was considered as significant and a *p*-value below 0.01 as very significant.

### 2.5. Ethical Considerations

All the biological and clinical records used in the present study were pseudonymized before analysis. In French laws, explicit consent of the patient is not needed for this type of analyses.

## 3. Results

### 3.1. Demographic Characteristics of the Cohort

During the period of inclusion, 296 samples could be selected according to the pre-specified inclusion criteria among the 639 samples (46.3%) diagnosed with *E. coli* at the Bacteriology laboratory of the Infectious Agents Department (CHU de Poitiers, France). This inclusion rate allowed the detection of a difference of 20%, with a confidence interval of 95% and a power of 90%.

Among the tested strains (*n* = 296), 114 were characterized as K1-positive strains (39.9%) and 182 (60.1%) as K1 negative strains.

### 3.2. K1-Antigen Distribution through Clinical Sample Type

All the clinical and biological characteristics of the included patients are summarized in Table 1. No difference could be observed in terms of sex or age between groups (*p* > 0.05). Samples were mainly urine samples (including less than one fifth of those using catheter) followed by blood culture and genital samples. No difference was observed comparing the time-to-positive culture (17.4 vs. 12.8 h) for blood culture (*p* > 0.05).

### 3.3. Distribution of AST Phenotypes among K1-Positive and K1-Negative Strains

All AST results were analyzed after the K1-phenotype characterization and during routine biological management of the samples. The profiles are summarized in Table 2.

For resistance to β lactam no difference could be observed between groups in prevalence for all susceptible strains, or for ampicillin, ticarcillin/piperacillin associated with beta-lactamase inhibitor, cephalosporin or carbapenem. Few ESBL-bearing *E. coli* were observed in this study without a difference between groups. Differences were observed regarding resistance to the ampicillin with the inhibitor and the ticarcillin, with higher prevalence in K1-negative strains compared to K1-positive strains (50.0% vs. 26.8% and 25.8% vs. 14.9% respectively; *p* < 0.05).

For resistance to quinolone, a higher proportion of resistant strains was observed in K1-negative strains (19.8% vs. 7.0%; *p* < 0.01), without any difference in terms of level of quinolone resistance (nalidixic acid only or associated with other quinolone; *p* > 0.05).

For resistance to Sulfamethoxazole—trimethoprim, a higher proportion of resistant strains was observed in K1-negative strains (30.2% vs. 12.3%; *p* < 0.01). On the contrary, differences could be observed between groups, in terms of resistance to aminoglycosides, furans and Fosfomycin (*p* > 0.05).

### 3.4. Proportion of Pluri-Resistant Strains According to K1-Antigen Status

After the exploration of antibiotic-resistance per family, antibiotic resistance was explored regarding pluri-resistance status (defined in this study as a strain resistant to at least two different classes among tested ones) (Table 3.). For this purpose, antibiotic molecules were categorized into six different classes (i.e., β lactam, aminoglycosides, quinolones, furanes, fosfomycins and sulfamethoxazoles-trimethoprim). Due to different recommendations for testing, not all classes were tested, and to overcome this possible bias, only β lactam, aminoglycosides, quinolones and sulfamethoxazole-trimethoprim were included for the class-by-class analysis when all classes were considered for numerical determination of pluri-resistance.

Regarding the number of classes that were incompletely susceptible (or resistant), a difference of proportion was observed for strains with altered susceptibility to at least two antibiotic classes (12.3% vs. 33.0%; *p* < 0.01). This was confirmed by the higher mean number of antimicrobial classes the bacterial strain was resistant to, in K1-negative strains compared to K1-positive ones (0.68 vs. 1.11; *p* < 0.01). This difference tends to decrease for a larger number of classes (13.7% for two classes; 3.2% for three classes and 2.7% for four classes), without statistical difference for strains with altered susceptibility to three out of four classes (*p* > 0.05).

## 4. Discussion

This study is the first, to the best of our knowledge, to analyze antibiotic resistance of *E. coli* isolated from clinical samples of all types, regarding their K1-antigen status in both hospitalized and ambulatory patients.

The association between virulence factors and antibiotic resistance has been studied for years, especially in medical science. For example, in *Staphylococcus aureus*, associations between methicillin-resistance (MRSA) and virulence factors are attentively observed, as they could be responsible for very severe disease. Strains of MRSA that spread in the community have demonstrated higher virulence and an expanded set of virulence factors (with high secretion level) compared to sensitive strains [22]. Reciprocally, hospital-acquired MRSA could be responsible for increased stimulation of immune cells leading to more severe consequences, especially when exposed to inappropriate antibiotic treatment [23]. In gram-negative bacteria, such as *Pseudomonas aeruginosa*, some studies have shown that oprD mutants (an efflux pump) were more virulent than their oprD+ counterparts in a mouse model of respiratory infections [24]. This null mutation commonly arises in clinical isolates during therapy and is associated with carbapenem resistance [25]. Infections with such isolates are associated with worse clinical outcomes, associating virulence and antibiotic resistance [26]. Finally, in *E. coli*, virulence factors located in the chromosome, such as aerobactin and fimbriae, are frequently absent in antibiotic-resistant isolates, contrarily to the ones resistant to tetracyclin (*tetA* and *tetB*-positive strains) [27,28,29,30]. On the other hand, blaCTX-M15-positive and blaOXA-2 positive UPEC isolates presented more *colV*, *colE2-E9*, *colIa-Ib*, *hlyA* and *csgA* genes and more *colM*, *colB*, *colE*, and *crl* genes respectively [31]. All in all, these results could highlight the difficulty to interpret results in such a highly diversified clinically-based population.

By design, non-expensive processes, e.g., agglutination assays, were applied to determine the possible interest of this determination in the routine management of the sample and of the isolated bacteria. Even if this process is not as precise as molecular biology determination processes, the description of the nature of the different genetic supports or mutations in previous studies has demonstrated the very low number of discrepant results of phenotypic agglutination compared to molecular biology. For example, the study by Kaczmarek et al. demonstrated a single discrepant result out of sixty-six tested samples, representing an exactitude of more than 98% for the agglutination assays when molecular biology is considered as a gold standard [32].

As described in Table 1, there was no over-representation of K1-positive *E. coli* prevalence compared to K1-negative strains in clinical samples. Many authors have demonstrated that the majority of neonatal meningitis cases are due to K1-positive *E. coli*, while other authors have reported that these particular strains are less frequent in vaginal tracts, in a similar proportion between pregnant and non-pregnant women [10,33,34,35]. Some authors have demonstrated, using DNA hybridization methods, that virulence patterns (including K1-capsules) of strains isolated from blood culture and cerebrospinal fluid were different from those of urogenital strains [36]. This observation is nowadays contested, due to similar representation, as in the present study, of K1-positive and K1 negative strains (40% and 60% approximately in the present study). Moreover, in the present study, a similar proportion of bacteremia due to *E. coli* were K1-positive or K1-negative. This observation is in contradiction with the previous observation of a difference of phenotypic characterization in blood culture for strains isolated in adults and children alike [12].

Furthermore, an insufficient number of puncture fluids was included to confirm/refute this observation, and the study is limited regarding this conclusion. Moreover, by design, and due to the clinical nature of this study and using phenotypic determination based on agglutination assays, (absence of) difference regarding the K1-antigen distribution between intestinal and extra-intestinal strains as suggested by previous publications cannot be confirmed [37].

In literal terms, “multi-drug resistant strains” (MDR) means "resistant to more than one antimicrobial agent". Nevertheless, and as stated in Magiorakos et al., this definition is not applied by Infection Control societies that consider MDR strains as gram-negative bacteria “resistant to three or more antimicrobial classes” [38]. In order to facilitate the reader’s understanding, and in order to highlight the difference between the accepted notion described in this article and the present manuscript, strains resistant to at least two classes are herein considered as pluri-resistant strains. Moreover, in the present manuscript, resistance to at least one molecule within an antibiotic class was used to indicate resistance to the entire category. While this impact could be considered as a limited and crude indicator, this approach has already been used by scientific networks such as the National Healthcare Safety Network, that define, for example, carbapenem-resistance in *Klebsiella* spp. as resistance to imipenem, meropenem, ertapenem or doripenem [39,40]. Both these comments support but also temper the findings of this article, calling for greater standardization of future studies.

K1-positive strains were observed as being less frequently associated with resistance modification of at least two different antimicrobial classes. In the present study, more than half of the strains tested for AST were susceptible to all antibiotics, without any difference between groups. This proportion has previously been suggested in some studies (60% in the study by Kaczmarek et al.) and demonstrated as higher than others (14.3% for Cisowska et al.) [32,41]. Up until now, no study has had a number of strains sufficient to explore the possible association of ESBL with K1-antigen. Nevertheless, and as expected, the results have not demonstrated a possible association of this plasmid-carried mechanism of antibiotic resistance with a genomic virulence factor such as K1-antigen capsule.

The present study used only validated processes for AST phenotype determination, based on the EUCAST process and recommendations and allowing for robust comparisons to other studies. Finally, regarding specific antimicrobial classes that were associated with K1-positive or -negative strains, the study demonstrated that in this cohort, K1-negative *E. coli* presented a phenotypic profile more frequently associated with chromosomal resistance (inhibitor-resistance TEM -IRT-, mutation in *gyrA/B* or *frxA/rdxA* implicated in resistance to quinolone and Trimethoprim—sulfamethoxazole respectively) and associated with K1-negative *E. coli* strains. In pediatric samples, Fujita et al. highlighted differences between *E. coli* strains regarding susceptibility to ampicillin, with higher susceptibility in K1 positive ones, which is supported by observations by Jamie et al. using urine sampled from pregnant women with a higher susceptible proportion in K1-positive strains. Both Cisowska et al. and Nolewasjka-Lasak et al. noted a similar representation of antibiotic resistance in urinary and vaginal/cervical strains respectively [41,42,43,44].

Compared to previous publications, such as Kaczmarek et al. in 2011, which focused on bacterial strains isolated from newborns, different AST phenotypes could be observed for penicillin associated with inhibitors with higher resistance in K1-negative strains, but there was no difference on second to fourth generation cephalosporin [32]. On the contrary, the difference for Trimethoprim—sulfamethoxazole and quinolone has not been previously demonstrated, except for cotrimoxazole, by Cisowska et al., on uro-pathogenic strains only [41]. Moreover, differences could be observed for strains insensitive to at least two antimicrobial classes. This observation is supported by the study of Cole et al. in which an inverse correlation between the total number of virulence factors and non-susceptible antibiotics appears [45]

All in all, these observations highlighted that K1-determination probably cannot be used when positive as a predictable marker of pre-emptive susceptibility.

## Figures and Tables

**Table 1:** Demographic characteristics and sample nature of the analyzed cohort.

Demographic or Biological Parameters			K1-Positive Strain (*n* = 114)	K1-Negative Strain (*n* = 182)
Sex (M/F)			31/83	49/133
Age (mean, SD)			61.9 (27.3)	61.3 (27.7)
Sample nature (*n*, % of the whole group)	Urine sample (all) (*n* = 235, 79.4% of the whole cohort)		90 (38.3)	145 (61.7)
		Urine sample (catheter) (*n* = 37) (% of the urine sample group)	16 (17.8)	21 (11.5)
	Blood sample (*n* = 31; 10.5% of the whole cohort)		12 (38.7)	19 (61.3)
	Genital sample (*n* = 11; 3.7% of the whole cohort)		4 (36.4)	7 (63.6)
		Genital sample (Female) (*n* = 10) (% of the genital sample group)	3 (75)	7 (100)
	Puncture fluid (*n* = 11; 3.7% of the whole cohort)		3 (27.3)	8 (72.7)
	Neonatal sample (Gastric lavage, Placenta) (*n* = 4; 1.4% of the whole cohort)		2 (50)	2 (50)
	Respiratory sample (*n* = 2; 0.7% of the whole cohort)		2 (100)	0 (0)
	Otolaryngology—Ophthalmology Sample (*n* = 2; 0.7% of the whole cohort)		1 (50)	1 (50)

**Table 2:** Comparison of the AST phenotypes per antibiotic molecules.

Antibiotic Susceptibility Testing Results		K1-Positive Strain (*n*; %)	K1-Negative Strain (*n*; %)	*p*-Value
β lactam (*n* = 296)				
	All susceptible (*n* = 123)	53 (46.5)	70 (38.5)	-
	≥1 Resistance (*n* = 173)	61 (53.5)	112 (61.5)	
	Ampicillin-resistant (*n* = 137/296; 46.3%)	45 (39.5)	92 (50.5)	-
	Ticarcillin-resistant (*n* = 133/296)	42 (26.8)	91 (50.0)	<0.05
	Resistance to Ampicillin + inhibitor (*n* = 64/296)	17 (14.9)	47 (25.8)	<0.05
	Resistance to Ticarcillin + inhibitor (*n* = 11/24)	3 (37.5)	8 (50.0)	-
	Resistance to Piperacillin + inhibitor (*n* = 24/296)	6 (5.3)	18 (9.9)	-
	ESBL (*n* = 8/296)	2 (1.8)	6 (3.3)	-
	Resistance to 2nd gen. Cephalosporin (*n* = 9/262)	2 (1.8)	7 (4.7)	-
	Resistance to 3rd gen. Cephalosporin (*n* = 7/296)	2 (1.8)	5 (2.7)	-
	Resistance to 4th gen. Cephalosporin (*n* = 2/24)	1 (11.1)	1 (6.7)	-
	Temocillin-resistant (*n* = 41/258)	16 (16.2)	25 (15.7)	-
	Aztreonam-resistant (*n* = 3/19)	1 (12.5)	2 (18.2)	-
	Carbapenem-resistant (*n* = 0/296; 0%)	0 (-)	0 (-)	-
Aminoglycoside-resistant (*n* = 11/296)				
	All	2 (1.75)	9 (4.94)	-
	G (*n* = 8)	2 (100)	6 (66.7)	
	GT (*n* = 1)	0 (-)	1 (11.1)	
	A (*n* = 2)	0 (-)	2 (22.2)	
Quinolones (*n* = 44/296)	All	8 (7.0)	36 (19.8)	<0.01
	Nalidixic acid only (*n* = 2) (% of the quinolone-resistant subgroup)	0 (0)	2 (100)	-
Quinolones-resistant (*n* = 44/296)		8 (7.0)	36 (19.8)	<0.01
Trimethoprim—sulfamethoxazole -resistant (*n* = 69/296)		14 (12.3)	55 (30.2)	<0.01
Furan-resistant (*n* = 1/275)		0 (-)	1 (0.6)	-
Fosfomycin-resistant (*n* = 4/259)		1 (1.0)	3 (1.9)	-

ESBL: extended-spectrum beta-lactamase; G: resistance to Gentamycin only; GT: resistance to Gentamycin and Tobramycin only; A: resistance to Amikacin.

**Table 3:** Comparison of the AST multi-resistant phenotypes.

Antibiotic Susceptibility Testing Results		K1-Positive Strain (*n* = 114)	K1-Negative Strain (*n* = 182)	*p*-Value
Pluri-resistance (*n*; %)		21 (18.4)	76 (41.8)	<0.01
Number of antimicrobial classes incompletely susceptible * (mean; SEM)		0.68 (0.07)	1.11 (0.08)	<0.01
Number of strains presenting X incompletely susceptible antimicrobial classes **				
	1 class (*n* = 104; 35.1%)	46 (40.4)	58 (31.9)	-
	2 classes (*n* = 56; 18.9%)	12 (10.5)	44 (24.2)	<0.01
	3 classes (*n* = 13; 4.4%)	2 (1.8)	11 (6.0)	- ***
	4 classes (*n* = 5; 1.7%)	0 (0)	5 (2.7)	-

* Classes were: B-lactam; aminoglycosides; Quinolones; Fosfomycin; Trimethoprim—sulfamethoxazole; furans; ** Classes were: B-lactam; aminoglycosides; Quinolones; Trimethoprim—sulfamethoxazole; *** statistical trends (*p* value < 0.1); SEM: Standard Error of the Mean.

## Data Availability

Not applicable.
